# A Hybrid Swin Transformer and Texture Feature Framework for Histopathological Classification of Paratuberculosis

**DOI:** 10.3390/bioengineering13091024

**Published:** 2026-09-02

**Authors:** Nokulunga Nhlapho, George Obaido, Ebenezer Esenogho

**Affiliations:** Center for Artificial Intelligence and Multidisciplinary Innovations, Department of Auditing, College of Accounting Sciences, University of South Africa, Pretoria 0002, South Africa; nhlapng@unisa.ac.za (N.N.); esenoe@unisa.ac.za (E.E.)

**Keywords:** paratuberculosis, histopathology, Swin Transformer, texture analysis, explainable AI, XGBoost

## Abstract

Paratuberculosis is a chronic infectious disease associated with substantial economic losses in livestock production. Histopathological examination remains an important diagnostic approach but can be time-consuming and dependent on specialist expertise, motivating the development of automated and interpretable image-classification methods. This study evaluated an explainable framework integrating a pretrained Swin-Tiny Transformer, handcrafted Gray-Level Co-occurrence Matrix (GLCM) and Local Binary Pattern (LBP) texture descriptors, and XGBoost classification for paratuberculosis histopathology image analysis. Following duplicate screening, 349 unique images comprising 199 MAP-positive and 150 MAP-negative samples were evaluated using stratified image-level five-fold cross-validation. Four model configurations were compared to assess the independent and incremental contributions of the learned and handcrafted feature representations. The standalone Swin-Tiny model achieved the highest mean ROC–AUC of 0.979±0.015, while the Swin-embedding XGBoost and hybrid Swin + GLCM/LBP + XGBoost models achieved mean ROC–AUC values of 0.977±0.016 and 0.977±0.017, respectively. The GLCM/LBP-only model achieved a mean ROC–AUC of 0.934±0.041, indicating that the handcrafted texture descriptors contained independently discriminative information but provided limited incremental value when combined with the Swin embeddings. Grad-CAM and XGBoost feature-importance analyses provided image-level and feature-level insights into model predictions. These findings demonstrate the effectiveness of Swin-Tiny representations for paratuberculosis histopathology image classification while highlighting the need for external validation using larger, independently sourced datasets.

## 1. Introduction

**Figure 1:**
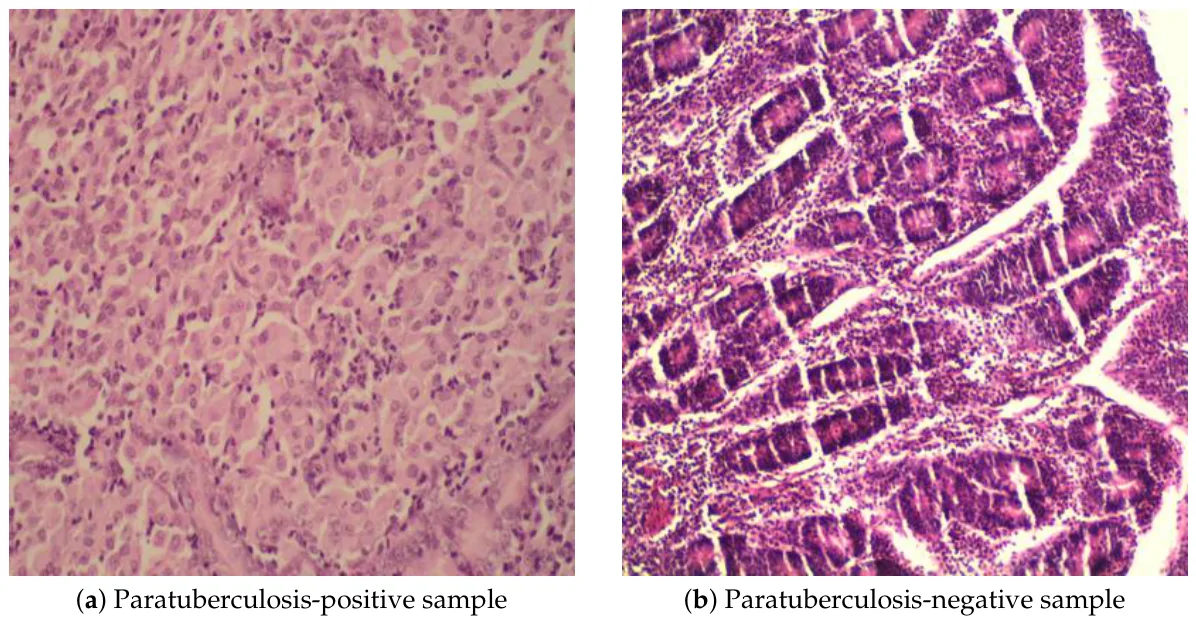
Representative histopathological images from the public paratuberculosis dataset: (**a**) MAP-positive sample and (**b**) MAP-negative sample. The displayed images are representative examples from the modeling image directories and contain no scale bars, annotations, or documentation borders. Images were subsequently subjected to the preprocessing procedures described in Section 2.

Paratuberculosis, also known as Johne’s disease, is a chronic infectious disease caused by *Mycobacterium avium* subspecies *paratuberculosis* (MAP) that primarily affects domestic and wild ruminants [1,2,3]. The disease is characterized by progressive intestinal inflammation, chronic diarrhea, weight loss, reduced productivity, and eventual deterioration, resulting in substantial economic losses and animal welfare concerns worldwide. Because infected animals can remain asymptomatic for extended periods while continuously shedding MAP, early and accurate diagnosis is essential for effective disease surveillance, herd management, and control programs aimed at reducing disease transmission and the associated economic burden [4,5,6]. Figure 1 presents representative histopathological tissue samples from paratuberculosis-positive and paratuberculosis-negative cases.

Histopathological examination remains an important diagnostic approach for evaluating paratuberculosis at the tissue level [7,8,9]. It enables direct visualization of granulomatous lesions, macrophage infiltration, alterations in lymphoid tissue, and other characteristic pathological manifestations associated with MAP infection. Histopathology also plays an important role in characterizing lesion patterns and understanding disease progression. However, manual assessment of histological slides is labor-intensive, requires considerable veterinary pathology expertise, and may be subject to inter-observer variability, particularly when lesions are subtle or heterogeneous. These limitations motivate the development of computational approaches capable of supporting pathologists through consistent and reproducible image interpretation [6,10,11]. Recent studies have further demonstrated the potential of machine-learning methods for quantitative veterinary histopathology and automated assessment of intestinal tissue, highlighting opportunities to improve analytical consistency and reduce observer-dependent variability [12].

Recent advances in artificial intelligence have demonstrated the potential of deep learning for automated medical and veterinary image analysis. Deep learning models have achieved strong performance in classification, detection, segmentation, and tissue-pattern recognition across diverse histopathological applications [9,13,14]. Recent reviews have also documented the expanding application of deep learning in veterinary diagnosis, including image-based disease detection, automated phenotyping, and clinical decision support [15]. Convolutional neural networks (CNNs) have historically dominated histopathological image analysis because of their ability to learn hierarchical representations directly from image data. However, within the field of paratuberculosis diagnosis, only a limited number of studies have investigated the automated analysis of histopathological tissue images.

For example, Sengoz et al. [16] developed a deep learning framework based on the VGG-16 convolutional neural network and demonstrated that contrast-limited adaptive histogram equalization (CLAHE) substantially improved classification performance, increasing the F1-score from 93% to 98%. To improve interpretability, Yigit et al. [17] proposed an explainable artificial intelligence framework for paratuberculosis diagnosis using ResNet and VGGNet architectures. More recently, machine-learning approaches have also been investigated for other aspects of Johne’s disease management. Imada et al. [18] evaluated tree-based machine-learning algorithms for predicting future Johne’s disease test outcomes from longitudinal testing data, demonstrating the broader potential of data-driven approaches for disease monitoring and herd-level decision support. Nevertheless, recent applications remain limited, and transformer-based hybrid approaches for paratuberculosis histopathological image classification have not been sufficiently investigated.

In another study, Wang et al. [19] developed a 3D-ResNet-based deep learning framework to distinguish nontuberculous mycobacterial lung disease from *Mycobacterium tuberculosis* using chest computed tomography images. The framework achieved an AUC of 0.86 on the test set, outperformed experienced radiologists, and automatically localized disease-related regions using class activation maps. Although the study focused on chest computed tomography rather than histopathology, it demonstrated that deep residual architectures combined with visual explanation methods can identify mycobacterial disease patterns while providing interpretable evidence to support clinical assessment.

Transformer-based architectures have emerged as powerful alternatives to CNNs because of their capacity to capture long-range spatial dependencies and contextual relationships extending beyond the local receptive fields of convolutional filters [20,21,22]. Recent research in computational histopathology has demonstrated the increasing use of vision transformers for image classification, tissue segmentation, cellular analysis, and outcome prediction because self-attention mechanisms can model contextual relationships across complex tissue regions [23,24]. Among transformer-based approaches, the Swin Transformer has attracted considerable attention because it introduces a hierarchical architecture based on shifted-window self-attention, enabling efficient modeling of local and global image structures while maintaining computational scalability [22,25]. Recent histopathological studies have further shown that transfer learning, data augmentation, image preprocessing, and appropriate model configuration are particularly important when adapting vision transformers to relatively small annotated datasets [26]. These characteristics make Swin-based architectures attractive for histopathological image analysis, where diagnostically relevant cellular and tissue patterns may occur across multiple spatial scales.

Despite the success of deep learning methods, histopathological classification remains challenging when datasets are relatively small, class distributions are imbalanced, or morphological differences between disease states are subtle [13,27]. In such situations, handcrafted texture descriptors may provide complementary information by explicitly quantifying local intensity distributions, spatial gray-level relationships, and tissue heterogeneity. Texture-analysis methods based on Gray-Level Co-occurrence Matrices (GLCM), Haralick statistics, and Local Binary Patterns (LBP) have been widely employed in biomedical image analysis and have demonstrated effectiveness in capturing diagnostically meaningful structural information that may not always be fully represented by deep neural features alone [20,28,29,30]. The integration of learned representations with handcrafted descriptors may therefore provide a useful strategy for evaluating whether explicit texture information contributes additional discriminatory value in limited-data histopathological settings.

In clinical and diagnostic settings, predictive performance alone is insufficient to support adoption. Automated systems should also provide transparent and interpretable explanations that enable domain experts to examine the rationale underlying model predictions. Black-box models may generate accurate outputs while relying on biologically irrelevant image regions, acquisition artifacts, or spurious correlations [31]. Consequently, explainable artificial intelligence (XAI) techniques have become increasingly important in medical image analysis. Methods such as Gradient-weighted Class Activation Mapping (Grad-CAM) and feature-importance analysis provide complementary insights into the image regions and feature representations that influence model decisions, thereby supporting transparency and expert validation [32,33]. Recent studies have also emphasized the importance of critically evaluating explanation methods in transformer-based histopathology because different approaches may vary in their ability to identify diagnostically meaningful tissue regions [34]. Table 1 summarizes the related work.

Motivated by these considerations, this study evaluates an explainable Swin Transformer and texture-feature framework for the binary classification of paratuberculosis in histopathological tissue images. Following duplicate screening and the exclusion of auxiliary images containing scale bars, annotations, or documentation artifacts, the final analytical cohort comprised 349 unique images, including 199 MAP-positive and 150 MAP-negative samples. Four model configurations were evaluated using identical stratified image-level five-fold cross-validation partitions: standalone end-to-end Swin-Tiny, GLCM/LBP features with XGBoost, Swin-Tiny embeddings with XGBoost, and a hybrid model combining Swin-Tiny embeddings with GLCM/LBP texture descriptors using XGBoost. This ablation design was used to assess the independent and incremental contributions of the learned and handcrafted feature representations. Grad-CAM and XGBoost feature-importance analyses were employed to provide complementary image-level and feature-level insights into model behavior.

The primary contributions of this work are threefold. The study:

1. develops a reproducible Swin Transformer-based framework for the binary classification of MAP-positive and MAP-negative histopathological tissue images using leakage-aware preprocessing and stratified image-level five-fold cross-validation;
2. conducts an ablation analysis comparing standalone Swin-Tiny, GLCM/LBP features with XGBoost, Swin-Tiny embeddings with XGBoost, and the complete hybrid configuration to evaluate the independent and incremental contributions of learned and handcrafted feature representations; and
3. integrates Grad-CAM and XGBoost feature-importance analyses to provide complementary image-level and feature-level interpretations of model behavior.

The remainder of this paper is organized as follows. Section 2 describes the dataset, preprocessing procedures, and feature-extraction process. Section 3 presents the Swin-Tiny and hybrid model architectures, training protocol, and evaluation methodology. Section 4 reports the experimental results and explainability analyses. Section 5 discusses the implications of the findings, while Section 6 presents the limitations of the current study. Section 7 concludes the paper and outlines directions for future research.

## 2. Materials and Methods

### 2.1. Dataset

This study utilized a publicly available histopathological image dataset for paratuberculosis introduced by Hananeh et al. [35]. The dataset comprises hematoxylin and eosin (H&E)-stained intestinal tissue images obtained from ruminants and manually classified by veterinary pathology experts. Histopathological examination is an established approach for identifying intestinal lesions associated with MAP infection, making these images suitable for evaluating image-based classification methods.

The original dataset contained 366 color histopathological images distributed across two diagnostic categories: 209 MAP-positive images and 157 MAP-negative images. Positive images represented tissue sections exhibiting histopathological characteristics associated with MAP infection, whereas negative images represented tissue samples without lesions classified as MAP-positive. According to the original dataset description, the specimens were collected from slaughterhouses located in Irbid, Amman, and Mafraq, Jordan, and were processed at the Jordan University of Science and Technology [35].

The images were organized into class-specific directories corresponding to the positive and negative diagnostic labels. Before model development, a dataset-integrity audit was conducted to identify duplicate files, visually altered copies, and potential non-biological image cues. Exact duplicates were screened using both cryptographic file hashes and hashes calculated from decoded pixel arrays. This procedure identified and removed 16 redundant exact-duplicate images. Perceptual similarity screening was subsequently performed to identify resized, recompressed, or visually modified versions of the same underlying image. One additional verified near-duplicate image was removed. Following duplicate screening, the final analytical cohort comprised 349 unique images, including 199 MAP-positive and 150 MAP-negative images. Table 2 summarizes the dataset-screening process.

Potential non-biological visual cues, including scale bars, measurement labels, annotations, documentation overlays, image borders, and related acquisition artifacts, were assessed before model development. Images identified as auxiliary or altered copies of an available clean histopathological image were excluded in favor of the corresponding original image.

The available dataset documentation, image filenames, and directory structure were also examined to determine whether information was available regarding animal identity, slide identity, specimen identity, scanner type, staining batch, and image magnification. Unique identifiers for individual animals, slides, and specimens were not provided. Consequently, images could not be grouped according to their biological source, and it was not possible to independently determine whether multiple images originated from the same animal, slide, or specimen. Cross-validation was therefore conducted at the image level after duplicate screening, as described in Section 3. Accordingly, the reported results represent internal image-level cross-validation performance and should not be interpreted as evidence of external diagnostic generalizability.

### 2.2. Data Preprocessing and Augmentation

Before model training, Reinhard stain normalization was applied to reduce variations in color appearance associated with differences in H&E staining and image acquisition. The normalization procedure was performed in the CIELAB color space by aligning the channel-wise means and standard deviations of each source image with a target color distribution derived exclusively from the outer training partition of each cross-validation fold. Specifically, the target means and standard deviations for the $L^{*}$, $a^{*}$, and $b^{*}$ channels were obtained by averaging the corresponding image-level statistics across the outer training images. The resulting fold-specific target statistics were calculated once and subsequently applied without modification to the outer training, inner validation, and outer test images within that fold. This procedure ensured that information from the held-out outer test partition was not used to define the stain-normalization parameters.

For each CIELAB channel, the normalized pixel values were calculated as

(1) $$C_{\mathrm{norm}}=\left(\frac{C-\mu_{\mathrm{source}}}{\sigma_{\mathrm{source}}+\epsilon }\right)\sigma_{\mathrm{target}}+\mu_{\mathrm{target}},$$

where *C* denotes the source $L^{*}$, $a^{*}$, or $b^{*}$ channel; $\mu_{\mathrm{source}}$ and $\sigma_{\mathrm{source}}$ denote the mean and standard deviation of the corresponding channel in the source image; $\mu_{\mathrm{target}}$ and $\sigma_{\mathrm{target}}$ denote the fold-specific target statistics derived from the outer training partition; and $\epsilon =10^{-6}$ is a small constant introduced to prevent division by zero. Following normalization, the $L^{*}$ channel was restricted to the range [0,100], while the $a^{*}$ and $b^{*}$ channels were restricted to [−128,127], before conversion back to the RGB color space.

For end-to-end Swin-Tiny fine-tuning, each Reinhard-normalized training image underwent random resized cropping to 224×224 pixels using a scale range of 0.80–1.00 and the default Torchvision aspect-ratio range of 3/4–4/3. Random horizontal and vertical flipping were each applied with a probability of 0.50. Additional augmentation comprised random rotation within $\pm 20^{\circ }$ and color jitter with brightness, contrast, saturation, and hue values of 0.15, 0.15, 0.10, and 0.02, respectively. The resulting RGB images were converted to tensors and normalized using the fixed ImageNet channel means of (0.485,0.456,0.406) and standard deviations of (0.229,0.224,0.225).

Reinhard normalization and ImageNet normalization served distinct purposes. Reinhard normalization reduced variations in histopathological color appearance, whereas ImageNet normalization standardized the image tensors according to the distribution expected by the ImageNet-pretrained Swin-Tiny backbone.

**Figure 2:**
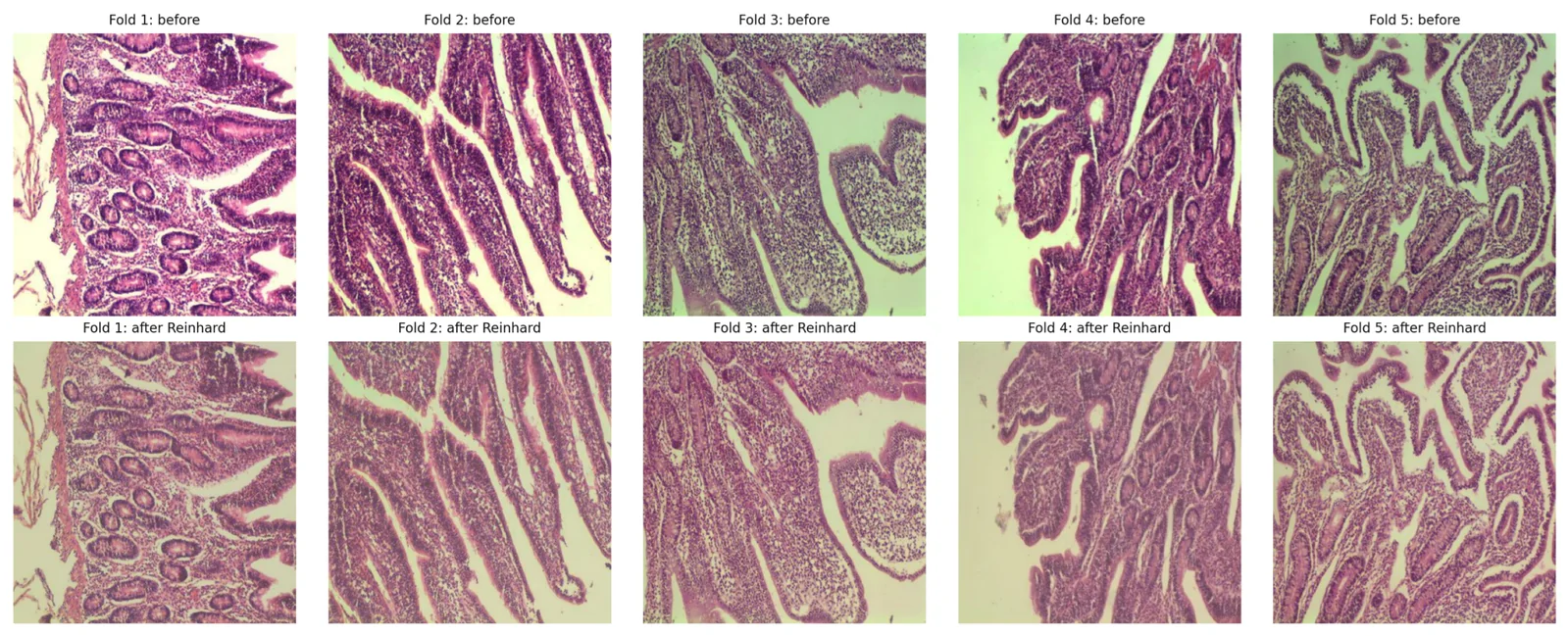
Representative histopathological images before and after Reinhard stain normalization across the five outer cross-validation folds. The (**upper row**) shows the original images, while the (**lower row**) shows the corresponding normalized images. For each outer fold, the target color statistics were estimated exclusively from the outer training partition and subsequently applied without modification to the outer training, inner validation, and outer test images within that fold.

Figure 2 presents representative images before and after fold-specific Reinhard stain normalization. The normalized images exhibit more consistent color distributions while retaining the underlying tissue morphology.

No random augmentation was applied to the inner validation or outer test partitions. These images underwent a deterministic sequence consisting of fold-specific Reinhard normalization, resizing to 224×224 pixels, conversion to tensors, and ImageNet normalization. This procedure ensured reproducible evaluation without stochastic transformations.

The same deterministic preprocessing sequence was also used when extracting Swin embeddings and handcrafted GLCM/LBP features for the XGBoost-based configurations. Accordingly, random augmentation was used only during end-to-end Swin-Tiny fine-tuning and was not applied when generating the fixed numerical feature representations used to train or evaluate the XGBoost classifiers. Table 3 summarizes the preprocessing and augmentation procedures.

### 2.3. Experimental Setup

The framework was implemented in Python 3.14.6 using PyTorch 2.13 Torchvision, timm, scikit-learn, and XGBoost. The Swin-Tiny Transformer was initialized with publicly available ImageNet-pretrained weights and fine-tuned for binary classification. Image preprocessing, including Reinhard stain normalization, spatial standardization, training-only augmentation, and ImageNet normalization, was performed as described in Section 2.2.

Model performance was evaluated using stratified five-fold cross-validation with a fixed random seed of 42. Because the public dataset did not provide unique identifiers for individual animals, histopathological slides, or tissue specimens, the folds were constructed at the image level after exact-duplicate and verified near-duplicate screening. The same outer-fold assignments were retained across all model configurations to enable paired performance comparisons under identical evaluation conditions. Each image appeared exactly once in the held-out outer test partition.

Within each outer fold, an inner validation partition was created exclusively from the corresponding outer training data. The inner validation partition was used for model selection and early stopping, whereas the outer test partition remained isolated until final evaluation. Fold-specific Reinhard target statistics and numerical feature-standardization parameters were estimated without using the outer test partition. This outer-training/inner-validation partitioning procedure was used to reduce information leakage during preprocessing, model selection, and performance evaluation.

The Swin-Tiny model was optimized using AdamW with an initial learning rate of $3\times 10^{-5}$, a batch size of 16, and a maximum of 50 training epochs. A weight decay of 0.05 was applied for regularization, and a cosine annealing schedule was used to adjust the learning rate during training. The optimization objective was a fold-specific weighted cross-entropy loss to account for the class distribution within each outer training partition. Early stopping was performed by monitoring the ROC–AUC on the inner validation partition, using a patience of 10 epochs and a minimum improvement threshold of $10^{-4}$. For each outer fold, the model checkpoint achieving the highest inner-validation ROC–AUC was retained for evaluation and deep-feature extraction.

Following fine-tuning, the retained Swin-Tiny model was used to extract a 768-dimensional embedding from each image. For the XGBoost-based configurations, the Swin embeddings and handcrafted texture descriptors were standardized separately using feature-wise Z-score transformations. The mean and standard deviation of each feature were estimated exclusively from the outer training partition and subsequently applied without modification to the corresponding inner validation and outer test data. The standardized feature blocks were then used independently or concatenated, depending on the model configuration.

The XGBoost classifier comprised 300 decision trees with a maximum tree depth of 3, a learning rate of 0.03, a subsampling ratio of 0.9, and a feature subsampling ratio of 0.9. Binary logistic loss was used as the optimization objective. Class imbalance was addressed within each outer fold by weighting the positive class according to the class distribution in the corresponding training partition. A fixed probability threshold of 0.50 was used to generate binary predictions and calculate threshold-dependent performance metrics.

To evaluate the independent and incremental contributions of the deep and handcrafted feature representations, four model configurations were assessed using the same cleaned dataset and identical outer-fold assignments: (1) standalone Swin-Tiny with its end-to-end classification head; (2) handcrafted GLCM and LBP texture descriptors with XGBoost; (3) Swin-Tiny embeddings with XGBoost; and (4) the complete hybrid configuration combining Swin-Tiny embeddings with GLCM and LBP texture descriptors using XGBoost. The four model configurations and their respective roles in the ablation analysis are summarized in Table 4. The comparison between the Swin-embedding XGBoost model and the complete hybrid model was designated as the primary ablation comparison because it isolated the incremental contribution of the handcrafted texture descriptors while retaining the same classifier and deep-feature representation.

All out-of-fold probabilities and predicted class labels were retained for performance estimation, confidence-interval calculation, and paired statistical comparisons. Computation was performed on CUDA-enabled hardware when available and otherwise defaulted to CPU execution. Random seeds were fixed for Python, NumPy, PyTorch, scikit-learn, and XGBoost to improve computational reproducibility.

### 2.4. Swin-Tiny Transformer Model

The primary deep learning architecture employed in this study was the Swin-Tiny Transformer, a hierarchical vision transformer that computes self-attention within local, non-overlapping windows and enables information exchange across neighboring windows through a shifted-window mechanism [22,25,39,40]. By restricting self-attention to local windows, the Swin architecture reduces computational complexity relative to global self-attention while retaining hierarchical representations of local and broader image structures. This property is particularly relevant to histopathological image analysis, in which diagnostically informative patterns may occur at different spatial scales.

Given an input image, the Swin-Tiny architecture initially partitions the image into non-overlapping patches, which are projected into feature embeddings and processed through successive hierarchical stages. Patch-merging operations progressively reduce the spatial resolution while increasing the feature dimensionality, enabling the network to learn representations ranging from localized tissue characteristics to broader histopathological structures.

Within each local window, multi-head self-attention is computed as

(2) $$\mathrm{Attention}\left(Q,K,V\right)=\mathrm{Softmax}\left(\frac{QK^{T}}{\sqrt{d_{k}}}+B\right)V,$$

where *Q*, *K*, and *V* denote the query, key, and value matrices, respectively; $d_{k}$ denotes the dimensionality of the query and key representations; and *B* represents the learnable relative position bias.

The output of each window-based self-attention block is obtained through layer normalization, residual connections, and a multilayer perceptron (MLP). The window-based multi-head self-attention operation is expressed as

(3) $$z^{l}=W\text{-}\mathrm{MSA}\left(\mathrm{LN}\left(z^{l-1}\right)\right)+z^{l-1},$$

followed by the MLP operation:

(4) $$z^{l+1}=\mathrm{MLP}\left(\mathrm{LN}\left(z^{l}\right)\right)+z^{l},$$

where LN denotes layer normalization and W-MSA denotes window-based multi-head self-attention. In subsequent blocks, shifted-window multi-head self-attention (SW-MSA) offsets the window partitioning scheme to facilitate information exchange across neighboring windows. The alternating use of W-MSA and SW-MSA enables the model to capture local tissue characteristics while progressively integrating contextual information across larger image regions [22].

The Swin-Tiny model was initialized using ImageNet-pretrained weights and adapted for binary classification by replacing the original classification head with a two-class output layer. All histopathological images were provided to the network at an input resolution of 224×224 pixels following the preprocessing procedures described in Section 2.2. The complete network was fine-tuned separately within each outer cross-validation fold using only the corresponding training partition. Model selection and early stopping were performed using the inner validation partition, while the outer test partition remained isolated until final evaluation.

For the standalone Swin-Tiny configuration, class probabilities were generated directly from the classification head of the retained fold-specific model. For the XGBoost-based configurations, the same fine-tuned Swin-Tiny model was used as a deep feature extractor. The representation obtained after the final feature-processing and global-pooling operations, but before the classification head, was retained as a 768-dimensional embedding for each image.

The extracted Swin-Tiny embeddings were standardized using feature-wise Z-score transformations fitted exclusively to the corresponding outer training partition, as described in Section 2.3. The standardized embeddings were subsequently evaluated either independently using XGBoost or concatenated with the standardized handcrafted GLCM and LBP texture descriptors for hybrid classification. Within each cross-validation fold, the same fine-tuned Swin-Tiny model and corresponding deep embeddings were used for both the Swin-feature + XGBoost and hybrid XGBoost configurations. This ensured that differences between these configurations reflected the addition of the handcrafted texture descriptors rather than differences in the underlying deep representation.

### 2.5. Texture Feature Extraction

To complement the deep representations learned by the Swin-Tiny Transformer, handcrafted texture descriptors were extracted using the Gray-Level Co-occurrence Matrix (GLCM) framework proposed by Haralick et al. [28,41] and Local Binary Pattern (LBP) analysis. GLCM descriptors characterize the spatial relationships between neighboring gray-level intensities, whereas LBP captures localized variations in texture by encoding the relative intensity patterns within a pixel neighborhood. The combined representation was used to evaluate whether handcrafted texture information provided complementary predictive information beyond the deep features learned by the Swin-Tiny model.

Texture features were extracted from the Reinhard-normalized images described in Section 2.2. Each RGB image was converted to grayscale before feature extraction. The grayscale intensities were quantized to 32 gray levels to reduce computational complexity and improve the stability of the co-occurrence estimates. The quantized intensity values ranged from 0 to 31 and were represented using an unsigned integer data type.

For each image, GLCMs were calculated using pixel distances of 1, 2, and 4 pixels and angular orientations of $0^{\circ }$, $45^{\circ }$, $90^{\circ }$, and $135^{\circ }$, corresponding to 0, π/4, π/2, and 3π/4 radians, respectively. The matrices were configured as symmetric and normalized so that each matrix represented a probability distribution. Five texture properties were calculated from each distance-angle combination: contrast, dissimilarity, homogeneity, energy, and correlation.

The contrast feature was calculated as:

(5) $$\mathrm{Contrast}=\sum_{i=0}^{N-1}\sum_{j=0}^{N-1}{\left(i-j\right)}^{2}P\left(i,j\right)$$

where P(i,j) denotes the normalized co-occurrence probability between gray levels *i* and *j*, and *N* denotes the number of quantized gray levels.

Dissimilarity was calculated as:

(6) $$\mathrm{Dissimilarity}=\sum_{i=0}^{N-1}\sum_{j=0}^{N-1}\left|i-j\right|P\left(i,j\right)$$

where larger values indicate greater local variation in gray-level intensities.

Homogeneity was calculated as:

(7) $$\mathrm{Homogeneity}=\sum_{i=0}^{N-1}\sum_{j=0}^{N-1}\frac{P(i,j)}{1+|i-j|}$$

with larger values indicating a greater concentration of co-occurrence probabilities near the principal diagonal.

Energy, which measures textural uniformity, was calculated as

(8) $$\mathrm{Energy}=\sum_{i=0}^{N-1}\sum_{j=0}^{N-1}P{\left(i,j\right)}^{2}$$

Correlation was calculated as:

(9) $$\mathrm{Correlation}=\sum_{i=0}^{N-1}\sum_{j=0}^{N-1}\frac{\left(i-\mu_{i}\right)\left(j-\mu_{j}\right)P\left(i,j\right)}{\sigma_{i}\sigma_{j}}$$

where $\mu_{i}$ and $\mu_{j}$ denote the marginal means and $\sigma_{i}$ and $\sigma_{j}$ denote the corresponding marginal standard deviations.

To obtain a compact representation that incorporated information across multiple spatial relationships, each GLCM property was aggregated across the evaluated distances and angular orientations. The mean and standard deviation of each property were calculated across all distance-angle combinations, resulting in 10 aggregated GLCM features. In addition, the mean entropy and maximum probability of the normalized co-occurrence matrices were retained, producing a final 12-dimensional GLCM feature vector.

Local texture information was additionally characterized using the rotation-invariant uniform LBP operator. LBP was calculated using 24 sampling points distributed over a circular neighborhood with a radius of 3 pixels. For a center pixel with intensity $g_{c}$, the LBP code was defined as:

(10) $${\mathrm{LBP}}_{P,R}=\sum_{p=0}^{P-1}s\left(g_{p}-g_{c}\right)2^{p}$$

where P=24 denotes the number of sampling points, R=3 denotes the neighborhood radius, $g_{p}$ represents the intensity of the *p*th neighboring pixel, and

(11) $$s\left(x\right)=\left\{\begin{array}{cc} 1, & x\geq 0,\\ 0, & x<0 \end{array}\right.$$

The resulting uniform LBP codes were summarized using a normalized 26-bin histogram. The 12 GLCM descriptors and 26 LBP histogram features were concatenated to form a 38-dimensional handcrafted texture representation. Before XGBoost classification, the texture features were standardized using feature-wise Z-score transformations fitted exclusively to the corresponding outer training partition, as described in Section 2.3. The same training-derived transformation was subsequently applied to the inner validation and outer test partitions.

### 2.6. Hybrid Swin-Texture XGBoost Model

Deep feature representations obtained from the fine-tuned Swin-Tiny Transformer were combined with handcrafted GLCM and LBP texture descriptors to construct the hybrid feature representation. For each image, the representation obtained after the final feature-processing and global-pooling operations of the Swin-Tiny model, but before its classification head, was retained as a 768-dimensional deep feature vector. Let

(12) $$f_{\mathrm{Swin}}=\left[f_{1},f_{2},\ldots ,f_{768}\right]\in R^{768}$$

denotes the Swin-Tiny feature vector. The handcrafted texture representation comprised 12 GLCM descriptors and a 26-bin LBP histogram, resulting in a 38-dimensional feature vector:

(13) $$f_{\mathrm{Texture}}=\left[t_{1},t_{2},\ldots ,t_{38}\right]\in R^{38}$$

Before feature fusion, the Swin-Tiny and handcrafted texture feature blocks were standardized separately using feature-wise Z-score transformations. For each feature, the mean and standard deviation were estimated exclusively from the corresponding outer training partition and subsequently applied without modification to the inner validation and outer test partitions. The standardized feature vectors are denoted by ${\tilde{f}}_{\mathrm{Swin}}$ and ${\tilde{f}}_{\mathrm{Texture}}$, respectively.

The final hybrid representation was formed by concatenating the standardized Swin-Tiny and texture feature vectors:

(14) $$z={\tilde{f}}_{\mathrm{Swin}}∥{\tilde{f}}_{\mathrm{Texture}}\in R^{806}$$

where ∥ denotes feature concatenation and z represents the resulting 806-dimensional hybrid feature vector. This fused representation was designed to combine the high-level semantic information captured by the Swin-Tiny Transformer with the spatial gray-level relationships and localized texture patterns characterized by the GLCM and LBP descriptors.

The hybrid feature vectors were subsequently used to train an XGBoost classifier [42]. XGBoost constructs an additive ensemble of decision trees. For the *i*th sample, the ensemble score at boosting iteration *K* is expressed as:

(15) $${\hat{s}}_{i}=\sum_{k=1}^{K}f_{k}\left(z_{i}\right)$$

where $f_{k}$ denotes the *k*th regression tree, *K* represents the total number of trees in the ensemble, and $z_{i}$ is the hybrid feature vector for sample *i*. Because binary logistic classification was used, the ensemble score was transformed into the estimated probability of MAP-positive classification using the logistic function:

(16) $${\hat{p}}_{i}=P\left(y_{i}=1|z_{i}\right)=\frac{1}{1+\exp(-{\hat{s}}_{i})}$$

A fixed probability threshold of 0.50 was used to obtain the predicted diagnostic class:

(17) $${\hat{y}}_{i}=\left\{\begin{array}{cc} 1, & {\hat{p}}_{i}\geq 0.50,\\ 0, & {\hat{p}}_{i}<0.50 \end{array}\right.$$

where ${\hat{y}}_{i}=1$ denotes a MAP-positive prediction and ${\hat{y}}_{i}=0$ denotes a MAP-negative prediction.

The XGBoost model was trained by minimizing a regularized objective function [42,43]:

(18) $$L=\sum_{i=1}^{N}l\left(y_{i},{\hat{s}}_{i}\right)+\sum_{k=1}^{K}\Omega \left(f_{k}\right)$$

where l(·) denotes the binary logistic loss between the observed class label $y_{i}$ and the predicted ensemble score ${\hat{s}}_{i}$, and $\Omega (f_{k})$ denotes the regularization term used to penalize model complexity. The regularization component controls the complexity of the individual decision trees and reduces the likelihood of overfitting.

The XGBoost classifier used 300 decision trees, a maximum tree depth of 3, a learning rate of 0.03, a subsampling ratio of 0.9, and a feature subsampling ratio of 0.9. Class imbalance was addressed within each outer cross-validation fold by weighting the positive class according to the class distribution in the corresponding training partition. The same XGBoost configuration was used for the GLCM/LBP-only, Swin-feature, and hybrid model configurations to support a controlled comparison of the different feature representations.

### 2.7. Evaluation Metrics

Model performance was evaluated using accuracy, precision, recall (sensitivity), specificity, F1-score, and the area under the receiver operating characteristic curve (ROC–AUC) [44,45]. Threshold-dependent metrics were calculated using a fixed classification probability threshold of 0.50. These metrics were derived from the confusion matrix, which comprised true positives (TP), true negatives (TN), false positives (FP), and false negatives (FN).

Classification accuracy measures the overall proportion of correctly classified samples and was calculated as:

(19) $$\mathrm{Accuracy}=\frac{TP+TN}{TP+TN+FP+FN}$$

Precision quantifies the proportion of samples predicted as MAP-positive that were correctly classified:

(20) $$\mathrm{Precision}=\frac{TP}{TP+FP}$$

Recall, also referred to as sensitivity or the true-positive rate, measures the proportion of MAP-positive samples correctly identified by the model:

(21) $$\mathrm{Recall}=\mathrm{Sensitivity}=\frac{TP}{TP+FN}$$

Specificity measures the proportion of MAP-negative samples correctly classified:

(22) $$\mathrm{Specificity}=\frac{TN}{TN+FP}$$

The F1-score represents the harmonic mean of precision and recall and was calculated as

(23) $$F_{1}=2\left(\frac{\mathrm{Precision}\times \mathrm{Recall}}{\mathrm{Precision}+\mathrm{Recall}}\right)$$

The receiver operating characteristic (ROC) curve describes the relationship between the true-positive rate and false-positive rate across all possible classification thresholds. The true-positive rate is equivalent to sensitivity and was calculated as:

(24) $$\mathrm{TPR}=\frac{TP}{TP+FN},$$

whereas the false-positive rate was calculated as

(25) $$\mathrm{FPR}=\frac{FP}{FP+TN}$$

The area under the ROC curve summarizes the ability of a model to discriminate between MAP-positive and MAP-negative images independently of a single classification threshold and is expressed as

(26) $$\mathrm{ROC}–\mathrm{AUC}=\int_{0}^{1}\mathrm{TPR}\left(\mathrm{FPR}\right)\;d\left(\mathrm{FPR}\right)$$

A ROC–AUC value of 0.50 indicates discrimination equivalent to random classification, whereas a value of 1.00 indicates perfect discrimination.

Performance metrics were calculated separately for each outer cross-validation fold and summarized using the mean and standard deviation across the five folds. In addition, the predicted probabilities generated for each image while it served in the corresponding held-out outer test partition were combined to produce pooled out-of-fold predictions. Each image therefore contributed exactly one held-out prediction per model configuration. The pooled out-of-fold predictions were used to generate the aggregate ROC curves, confusion matrices, and overall performance estimates.

To quantify uncertainty, 95% confidence intervals were estimated from the pooled out-of-fold predictions using stratified bootstrap resampling with 2000 iterations. During each bootstrap iteration, images were resampled with replacement while preserving representation from both diagnostic classes. Confidence intervals were calculated using the 2.5th and 97.5th percentiles of the resulting bootstrap distributions. Because unique animal, slide, and specimen identifiers were unavailable, bootstrap resampling was performed at the image level.

Paired statistical comparisons were conducted using predictions generated for the same held-out images. The primary comparison evaluated the Swin-feature + XGBoost model against the hybrid Swin + GLCM/LBP XGBoost model because these configurations used the same Swin embeddings, XGBoost classifier, preprocessing procedures, cross-validation folds, and test images, differing only in the inclusion of the handcrafted texture descriptors. The difference in ROC–AUC between the two models was evaluated using paired bootstrap resampling, and a 95% confidence interval was calculated for the paired ROC–AUC difference. A difference was not considered statistically supported when the corresponding confidence interval included zero.

McNemar’s test was additionally used to compare paired binary classification outcomes at the fixed probability threshold of 0.50. The test evaluated the discordant predictions between two models, including observations correctly classified by one model but incorrectly classified by the other. Statistical significance was assessed using a two-sided significance level of α=0.05.

To provide transparency regarding classification outcomes, TP, TN, FP, and FN values were reported for each model configuration. All confidence intervals and statistical comparisons were calculated using predictions obtained exclusively from the held-out outer test partitions.

### 2.8. Explainability Methods

To improve model transparency and examine the information used during classification, complementary explainability analyses were conducted using Gradient-weighted Class Activation Mapping (Grad-CAM) [46] and XGBoost feature-importance analysis [47]. Grad-CAM was used to generate image-level visual explanations for the standalone Swin-Tiny model by identifying spatial regions associated with the predicted class. XGBoost feature importance was used to provide model-level explanations by quantifying the relative contributions of the Swin-derived and handcrafted texture features to the tree-based classification models.

For Grad-CAM, activation maps and class-specific gradients were obtained from the retained fold-specific Swin-Tiny models. For a target class *c*, the importance weight associated with feature map *k* was calculated as:

(27) $$\alpha_{k}^{c}=\frac{1}{Z}\sum_{i}\sum_{j}\frac{\partial y^{c}}{\partial A_{ij}^{k}},$$

where $A_{ij}^{k}$ denotes the activation at spatial location (i,j) in feature map *k*, $y^{c}$ denotes the model score for class *c*, and *Z* represents the total number of spatial locations in the feature map.

The class-specific Grad-CAM heatmap was subsequently calculated as

(28) $$L_{\mathrm{GradCAM}}^{c}=\mathrm{ReLU}\left(\sum_{k}\alpha_{k}^{c}A^{k}\right),$$

where the rectified linear unit retains positive contributions associated with the target class. The resulting activation maps were normalized, resized to the dimensions of the corresponding histopathological images, and superimposed on the images to facilitate visual interpretation. Regions with higher activation intensity indicate image areas that contribute more strongly to the model score for the selected class.

Grad-CAM visualizations were generated using the retained Swin-Tiny checkpoint corresponding to the outer cross-validation fold in which each selected image served as a held-out test observation. This procedure ensured that the visual explanations were derived from models that had not been trained using the corresponding images. Representative correctly classified and misclassified MAP-positive and MAP-negative images were examined to assess whether the model emphasized tissue regions rather than image borders, annotations, or other non-biological visual cues.

For the XGBoost-based configurations, feature importance was quantified using gain, which represents the improvement in the model objective attributable to decision-tree splits involving a given feature. Let T denote the set of trees in the XGBoost ensemble. The cumulative gain associated with feature *f* was expressed as:

(29) $$I\left(f\right)=\sum_{t\in T}\sum_{s\in S_{t}\left(f\right)}\Delta L\left(s\right),$$

where $S_{t}\left(f\right)$ denotes the set of decision nodes in tree *t* that use feature *f*, and ΔL(s) represents the improvement in the objective function resulting from split *s*. Features with larger gain values exerted a greater influence on the fitted XGBoost decision rules.

To facilitate comparison across feature types, individual feature-gain values were additionally aggregated according to their feature family. Features were grouped as Swin-Tiny embeddings, GLCM descriptors, or LBP descriptors. For a feature family F, its relative contribution was calculated as

(30) $$C\left(F\right)=\frac{\sum_{f\in F}I\left(f\right)}{\sum_{f\in A}I\left(f\right)},$$

where A denotes the complete set of features included in the fitted model. The resulting proportions were used to assess the relative contribution of the deep and handcrafted feature families within the hybrid classifier.

The feature-importance analysis was interpreted together with the ablation results because importance values alone do not establish that a feature family improves predictive performance. In addition, Grad-CAM and XGBoost gain represent model-specific explanatory measures and should not be interpreted as direct evidence of pathological causality or independent biological validation.

## 3. Hybrid Swin Transformer and Texture Feature Framework

This study developed a hybrid image-classification framework integrating a pretrained Swin-Tiny Transformer, handcrafted GLCM and LBP texture descriptors, and an XGBoost classifier for the automated classification of paratuberculosis from histopathological tissue images. The framework was designed to evaluate whether handcrafted texture characteristics provide additional predictive information beyond the deep representations learned by the Swin-Tiny Transformer. The overall pipeline comprises six principal stages: dataset integrity screening, image preprocessing and augmentation, deep and handcrafted feature extraction, feature standardization and fusion, classification and probability estimation, and model explainability.

**Figure 3:**
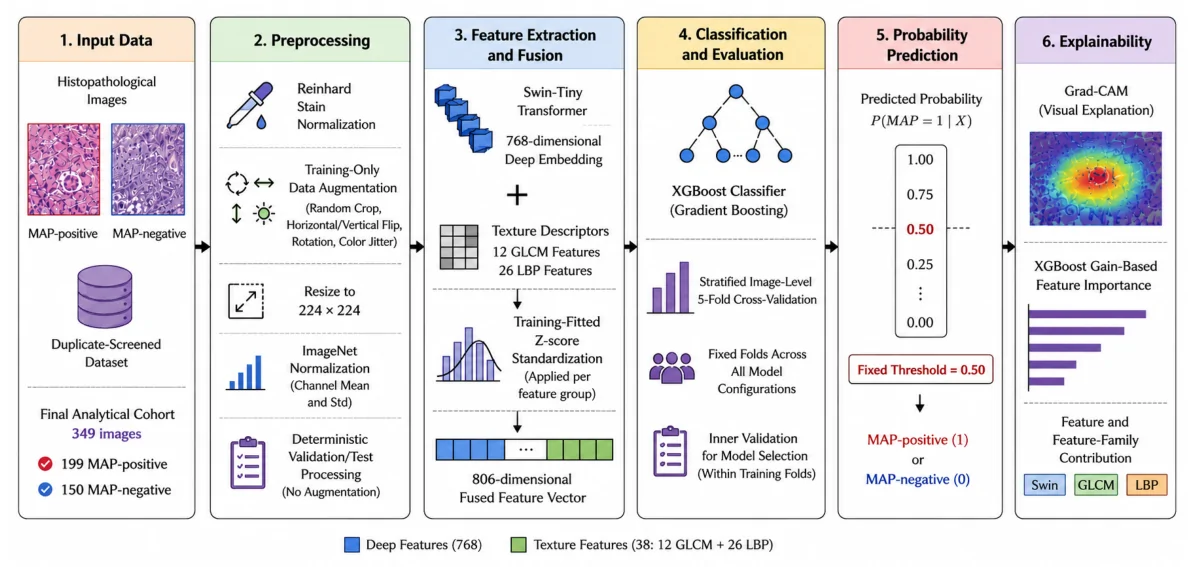
Overview of the proposed hybrid framework for automated classification of paratuberculosis from histopathological tissue images. The workflow includes dataset integrity screening, fold-specific Reinhard stain normalization, image preprocessing, Swin-Tiny deep-feature extraction, GLCM and LBP texture-feature extraction, feature standardization and fusion, XGBoost classification, and complementary explainability analyses using Grad-CAM and feature importance.

Figure 3 presents the end-to-end workflow of the proposed framework. The pipeline transforms histopathological tissue images into estimated probabilities of MAP-positive classification while providing image-level and feature-level explanations to facilitate interpretation of the model predictions.

The workflow begins with histopathological tissue images labelled as MAP-positive or MAP-negative. Before model development, the dataset was screened for exact duplicates, visually altered copies, and potential non-biological image cues. Following the removal of redundant exact duplicates and one verified near-duplicate, the final analytical cohort comprised 349 unique images. Because unique identifiers for individual animals, histopathological slides, and tissue specimens were unavailable, model evaluation was conducted using stratified image-level five-fold cross-validation. Identical outer-fold assignments were retained across all model configurations to support paired comparisons under consistent evaluation conditions.

Within each outer cross-validation fold, Reinhard stain normalization was applied using target color statistics derived exclusively from the corresponding outer training partition. The resulting images were subsequently processed according to the requirements of the ImageNet-pretrained Swin-Tiny architecture. During model training, random resized cropping to 224×224 pixels, horizontal and vertical flipping, rotation, and color jittering were applied to increase image diversity and reduce overfitting. Data augmentation was restricted to the training partition. Inner validation and outer test images underwent deterministic resizing without random augmentation. Following conversion to tensors, all images were standardized using the fixed ImageNet channel means and standard deviations.

Each preprocessed image was passed through the fine-tuned Swin-Tiny Transformer to learn hierarchical representations of local tissue characteristics and broader histopathological context. The representation obtained before the classification head was retained as a 768-dimensional deep-feature embedding. In parallel, handcrafted texture characteristics were extracted from the Reinhard-normalized images using GLCM and LBP analysis. The handcrafted representation comprised 12 GLCM descriptors and a normalized 26-bin LBP histogram, resulting in a 38-dimensional texture-feature vector.

For the hybrid configuration, the Swin-Tiny and handcrafted texture feature blocks were standardized separately using feature-wise Z-score transformations fitted exclusively to the corresponding outer training partition. The standardized 768-dimensional Swin embedding was then concatenated with the standardized 38-dimensional GLCM/LBP representation to produce an 806-dimensional fused feature vector. The fused representation was supplied to an XGBoost classifier, which modeled nonlinear relationships among the deep and handcrafted features and generated an estimated probability of MAP-positive classification. A fixed probability threshold of 0.50 was used to assign MAP-positive or MAP-negative class labels.

To evaluate the independent and incremental contributions of the feature representations, four model configurations were assessed using the same cleaned dataset and identical outer-fold assignments: standalone Swin-Tiny, GLCM/LBP features with XGBoost, Swin-Tiny embeddings with XGBoost, and the complete hybrid model combining Swin-Tiny embeddings with GLCM/LBP features using XGBoost. This ablation design enabled the contribution of the handcrafted texture representation to be evaluated independently while controlling for the underlying deep representation and classifier.

Model transparency was examined using complementary image-level and model-level explainability methods. Grad-CAM was applied to the retained fold-specific Swin-Tiny models to identify image regions associated with the predicted class. XGBoost gain-based feature importance was used to quantify the relative influence of individual features and the aggregate contributions of the Swin, GLCM, and LBP feature families. These analyses were used to characterize the information emphasized by the fitted models and were interpreted together with the ablation results. The resulting explanations represent model-specific patterns and were not interpreted as evidence of pathological causality or external clinical validity.

## 4. Results

### 4.1. Comparative Performance and Ablation Analysis

To assess the independent and incremental contributions of the deep and handcrafted feature representations, four model configurations were evaluated using identical stratified image-level five-fold cross-validation partitions: standalone end-to-end Swin-Tiny, GLCM/LBP features with XGBoost, Swin-Tiny embeddings with XGBoost, and the complete hybrid model combining Swin-Tiny embeddings with GLCM/LBP features using XGBoost.

Table 5 summarizes the mean performance and standard deviation obtained across the five outer cross-validation folds. The standalone Swin-Tiny model achieved the highest mean ROC–AUC of 0.979±0.015, together with an accuracy of 0.920±0.036 and an F1-score of 0.930±0.029. The Swin-embedding XGBoost model achieved the highest mean precision of 0.959±0.039 and, together with the hybrid model, obtained the highest mean accuracy of 0.928 and specificity of 0.947.

**Figure 4:**
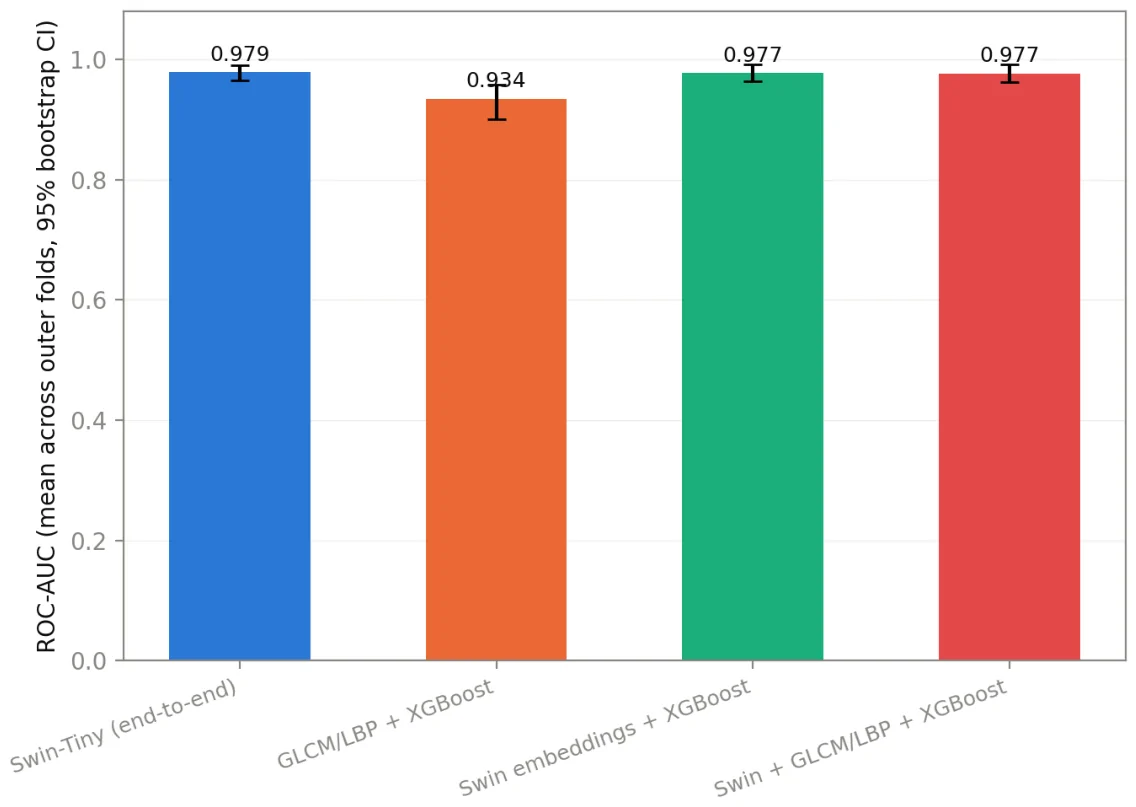
Comparison of mean ROC–AUC values across the four model configurations evaluated using stratified image-level five-fold cross-validation. Error bars represent 95% bootstrap confidence intervals. The standalone Swin-Tiny model achieved the highest mean ROC–AUC, while the Swin-embedding XGBoost and hybrid Swin + GLCM/LBP + XGBoost configurations produced comparable performance.

Figure 4 provides a visual comparison of the mean ROC–AUC values and corresponding 95% bootstrap confidence intervals across the four model configurations. The substantial overlap among the confidence intervals of the three Swin-based configurations indicates comparable discriminatory performance, whereas the GLCM/LBP-only model achieved a lower mean ROC–AUC.

The GLCM/LBP-only XGBoost model achieved a mean ROC–AUC of 0.934±0.041, indicating that the handcrafted texture descriptors contained independently discriminative information for distinguishing MAP-positive from MAP-negative tissue images. However, its mean accuracy, specificity, and F1-score were lower than those of all configurations incorporating Swin-Tiny representations. This finding indicates that the learned deep representations captured stronger discriminatory information than the handcrafted texture descriptors when the two feature families were evaluated independently.

The Swin-embedding XGBoost model and the complete hybrid model produced nearly identical mean cross-validation performance. Both configurations achieved a mean accuracy of 0.928, recall of 0.915, specificity of 0.947, F1-score of 0.936, and ROC–AUC of 0.977. The addition of the 38-dimensional GLCM/LBP representation therefore did not improve mean cross-validation performance beyond that obtained using the Swin-Tiny embeddings alone. The standalone end-to-end Swin-Tiny model retained the highest mean ROC–AUC, although the differences among the three Swin-based configurations were small.

The ablation findings show that most of the predictive performance was associated with the Swin-Tiny representation. Although the handcrafted GLCM/LBP features exhibited independent discriminatory capability, their addition to the Swin embeddings did not improve the mean ROC–AUC or the other evaluated performance metrics. Statistical comparisons based on pooled out-of-fold predictions are presented in Section 4.3.

### 4.2. Pooled Out-of-Fold Classification Performance

To complement the fold-level performance estimates reported in Table 5, predictions from the held-out outer test partitions were combined to obtain pooled out-of-fold classification outcomes. Each image contributed exactly one prediction generated by a model that had not been trained using that image. The pooled predictions therefore provide an aggregate assessment of classification performance across the complete analytical cohort while preserving the separation between training and held-out test data.

**Figure 5:**
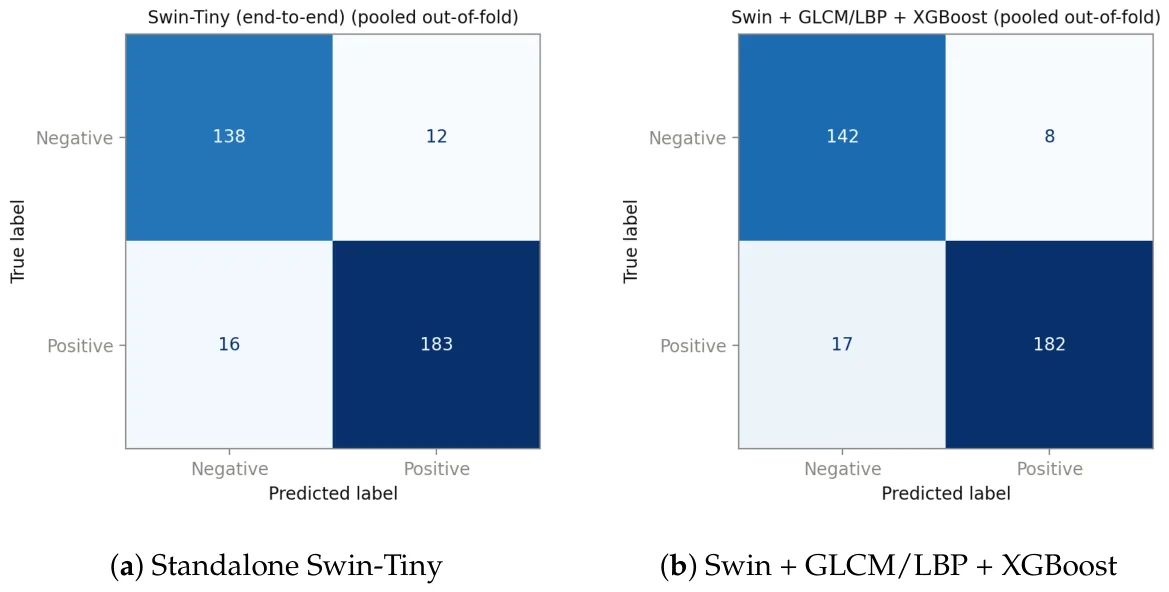
Pooled out-of-fold confusion matrices for the standalone Swin-Tiny and hybrid Swin + GLCM/LBP + XGBoost models at the fixed classification threshold of 0.50. Each image contributed one prediction obtained from the outer test fold in which it was evaluated.

Figure 5 presents the pooled out-of-fold confusion matrices for the standalone Swin-Tiny and hybrid Swin + GLCM/LBP + XGBoost models at the fixed probability threshold of 0.50.

As shown in Figure 5, the standalone Swin-Tiny model correctly classified 138 of the 150 MAP-negative images and 183 of the 199 MAP-positive images, yielding 12 false-positive and 16 false-negative predictions. The hybrid model correctly classified 142 MAP-negative images and 182 MAP-positive images, yielding 8 false-positive and 17 false-negative predictions.

Relative to the standalone Swin-Tiny model, the hybrid configuration reduced the number of false-positive predictions from 12 to 8 while producing one additional false-negative prediction. Thus, the hybrid model demonstrated a modest shift toward improved identification of MAP-negative samples rather than a consistent improvement across all classification outcomes. These findings are consistent with the fold-level results in Table 5, which showed comparable overall performance among the Swin-based configurations.

### 4.3. Statistical Comparison of Model Configurations

To determine whether the observed differences among the model configurations were statistically supported, paired comparisons were performed using the pooled out-of-fold predictions. Differences in ROC–AUC were evaluated using paired bootstrap resampling with 2000 iterations, while differences in threshold-based classification outcomes were assessed using McNemar’s exact test at the fixed probability threshold of 0.50. Because all model configurations used identical outer-fold assignments, predictions could be compared on the same held-out images.

The primary comparison evaluated the Swin-embedding XGBoost model against the complete hybrid Swin + GLCM/LBP + XGBoost model. These configurations used the same Swin-Tiny embeddings, XGBoost classifier, cross-validation partitions, and held-out test images and differed only in the inclusion of the 38-dimensional handcrafted texture representation. Therefore, this comparison directly assessed the incremental contribution of the GLCM and LBP features. The results of the primary and secondary statistical comparisons are summarized in Table 6.

As shown in Table 6, the hybrid model produced a pooled ROC–AUC difference of −0.00084 relative to the Swin-embedding XGBoost model. The corresponding 95% confidence interval, ranging from −0.00214 to 0.00020, included zero, indicating that the addition of the GLCM/LBP features did not produce a statistically supported improvement in discriminatory performance. The magnitude of the difference was also negligible, suggesting no practically meaningful change in ROC–AUC.

At the fixed probability threshold of 0.50, the Swin-embedding and hybrid XGBoost models produced identical aggregate confusion-matrix counts, with 142 true-negative, 8 false-positive, 17 false-negative, and 182 true-positive predictions. McNemar’s exact test yielded p=1.000, indicating no evidence of a difference in paired classification correctness. Although the aggregate confusion-matrix counts were identical, one image that was incorrectly classified by the Swin-embedding model was correctly classified by the hybrid model, while one image that was correctly classified by the Swin-embedding model was incorrectly classified by the hybrid model. These opposing changes resulted in no net difference in the aggregate classification outcomes.

The comparison between the standalone Swin-Tiny and hybrid models similarly showed no statistically supported difference. The hybrid-minus-Swin ROC–AUC difference was −0.00050, with a 95% confidence interval of [−0.0105,0.0091], while McNemar’s exact test yielded p=0.549. Thus, the hybrid configuration did not demonstrate improved discriminatory or threshold-based classification performance relative to the end-to-end Swin-Tiny model.

In contrast, the hybrid model significantly outperformed the GLCM/LBP-only XGBoost model. The pooled ROC–AUC difference was 0.0460, with a 95% confidence interval of [0.0248,0.0709], which excluded zero. McNemar’s exact test also indicated a significant difference in classification outcomes (p<0.001). This finding demonstrates that the inclusion of the Swin-Tiny representation substantially improved performance relative to the handcrafted texture representation alone.

The statistical analyses indicate that the Swin-Tiny representation accounted for the principal predictive improvement within the hybrid framework. Although the GLCM/LBP descriptors demonstrated independent discriminatory capability, their addition to the Swin embeddings did not provide a statistically supported or practically meaningful incremental benefit.

### 4.4. Deep and Handcrafted Feature Representations

**Figure 6:**
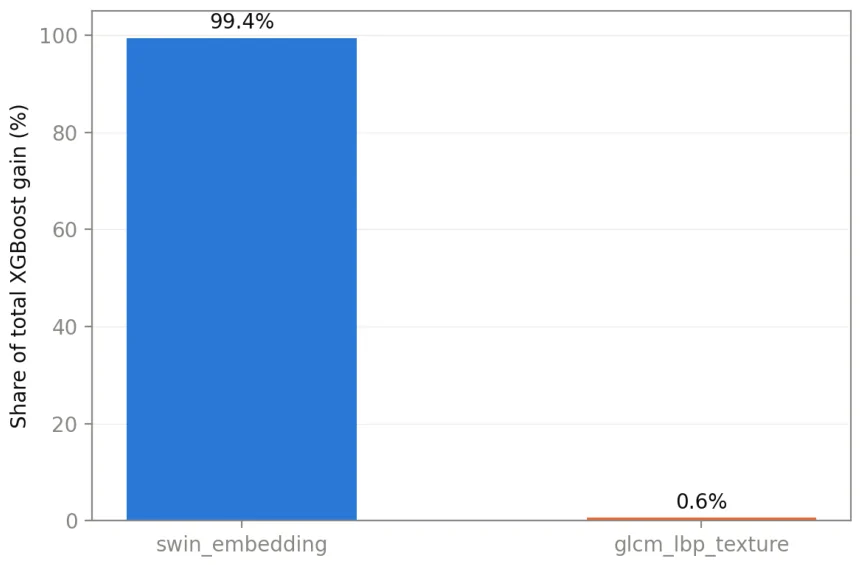
Aggregate gain-based contribution of the Swin-Tiny embedding and handcrafted GLCM/LBP feature families in the hybrid XGBoost model across the five outer cross-validation folds. Swin-derived features accounted for 99.4% of the total gain, whereas the combined GLCM/LBP texture representation accounted for 0.6%.

To further examine the relative contributions of the deep and handcrafted feature representations, gain-based feature importance was aggregated across the five outer-fold XGBoost models. Individual features were grouped according to their source as either Swin-Tiny embeddings or handcrafted GLCM/LBP texture descriptors. The resulting feature-family contributions are presented in Figure 6.

As shown in Figure 6, the Swin-Tiny embedding features accounted for 99.4% of the aggregate XGBoost gain, whereas the combined GLCM/LBP texture descriptors accounted for 0.6%. Although the handcrafted representation comprised 38 features, including 12 GLCM descriptors and 26 LBP histogram features, its aggregate contribution to the fitted decision-tree models was limited relative to that of the 768-dimensional Swin representation.

**Figure 7:**
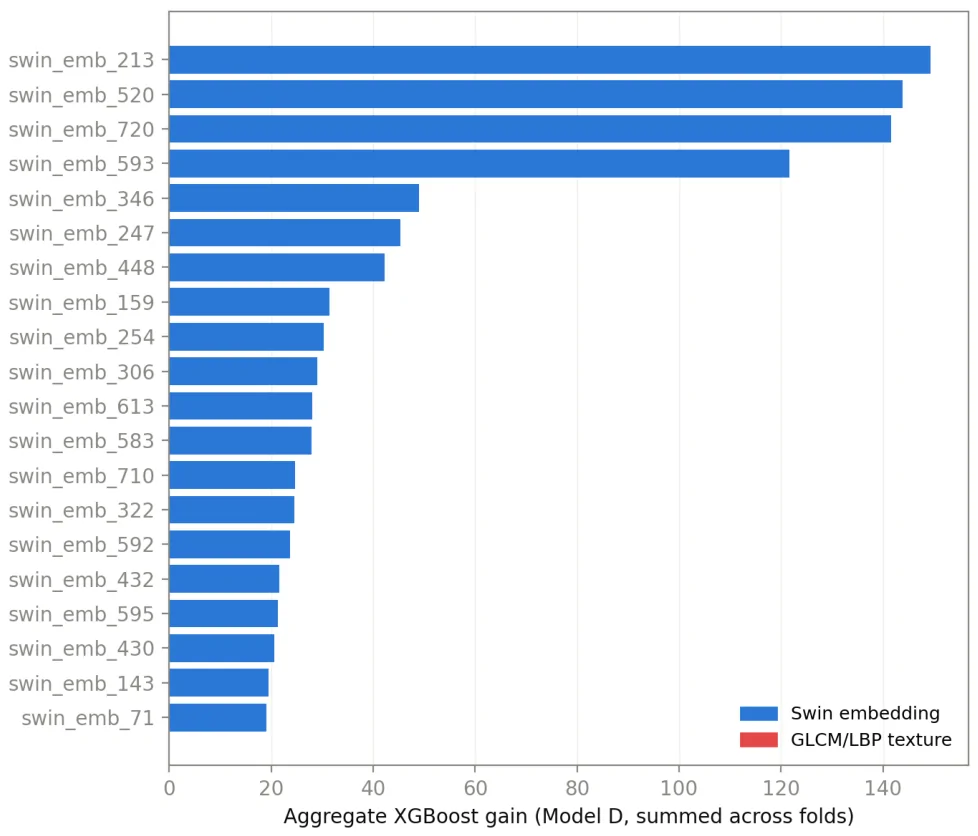
Twenty highest-ranked individual features in the hybrid XGBoost model based on aggregate gain across the five outer cross-validation folds. All features shown were derived from the Swin-Tiny embedding space; no individual GLCM or LBP feature appeared among the 20 highest-ranked predictors.

The ranking of individual features provided further evidence that the hybrid classifier relied predominantly on the learned deep representation. Figure 7 presents the 20 highest-ranked features according to aggregate XGBoost gain across the five outer folds.

As illustrated in Figure 7, all 20 highest-ranked features originated from the Swin-Tiny embedding space. No individual GLCM or LBP descriptor appeared among the highest-ranked predictors. This pattern was consistent with the feature-family analysis and indicated that the XGBoost classifier relied primarily on the learned deep representations when both feature families were available.

These findings provide a model-level explanation for the ablation and statistical results. The GLCM/LBP-only model achieved a mean ROC–AUC of 0.934±0.041, demonstrating that the handcrafted texture representation contained independently discriminative information. However, the limited gain attributed to the texture features in the hybrid model indicates that much of this information was already represented within the Swin-Tiny embedding space. Consequently, adding the handcrafted descriptors did not produce a measurable improvement over the Swin-embedding XGBoost configuration.

The gain-based importance values should be interpreted as measures of feature use within the fitted XGBoost models rather than as direct evidence of biological relevance. Furthermore, differences in the dimensionality of the feature families should be considered when interpreting aggregate importance because the Swin representation contained 768 features, whereas the handcrafted texture representation contained 38 features. Nevertheless, the absence of GLCM/LBP descriptors among the highest-ranked predictors, together with their limited aggregate gain and the results of the paired ablation analysis, consistently indicates that the handcrafted texture features provided little incremental predictive value in the present dataset.

### 4.5. Model Explainability

Grad-CAM was used to examine the spatial regions associated with the predictions generated by the standalone Swin-Tiny model. Each activation map was produced using the retained fold-specific model corresponding to the outer test partition in which the selected image was evaluated. Therefore, the visual explanations were generated from models that had not been trained using the corresponding images.

**Figure 8:**
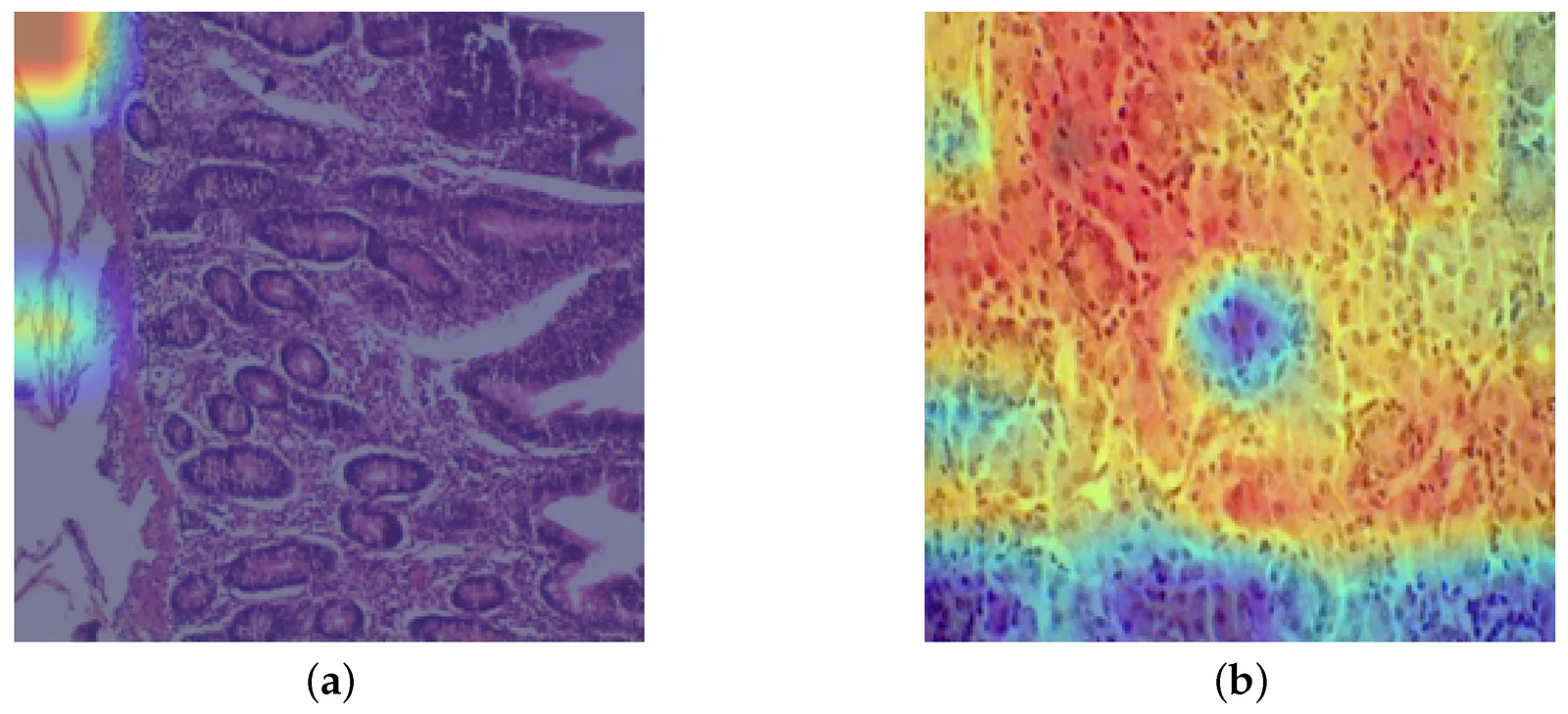
Representative Grad-CAM visualizations generated for correctly classified held-out histopathological images using the corresponding fold-specific standalone Swin-Tiny models. Panel (**a**) shows a correctly classified MAP-negative image, whereas panel (**b**) shows a correctly classified MAP-positive image. Warmer regions indicate image areas associated with greater contributions to the predicted class score, while cooler regions indicate comparatively lower contributions.

Figure 8 presents representative Grad-CAM visualizations for correctly classified MAP-negative and MAP-positive histopathological images. The activation maps were superimposed on the corresponding input images, with warmer regions indicating image areas associated with greater contributions to the predicted class score and cooler regions indicating comparatively lower contributions.

As shown in the figure, the activation patterns differed between the selected MAP-negative and MAP-positive examples. In the MAP-negative image, the strongest responses were localized to selected tissue regions and portions of the image periphery. In the MAP-positive image, activation was more broadly distributed across the tissue field, with several regions exhibiting comparatively strong responses. These examples indicate that the model used spatially distributed image information rather than relying on a single fixed image location.

The activation maps provide qualitative insight into the image regions associated with the model predictions but do not establish that the highlighted areas correspond to specific MAP-associated histopathological lesions. Because the activated regions were not independently evaluated against pathologist-provided region-level annotations, the visualizations should be interpreted as post hoc explanations of model behavior rather than evidence of pathological causality or diagnostic validity.

The Grad-CAM analysis complements the feature-level results presented in Section 4.4. Whereas Grad-CAM provides spatial information regarding image regions associated with the standalone Swin-Tiny predictions, the gain-based XGBoost analysis indicates that the hybrid classifier relied predominantly on Swin-derived embedding features. Together, these analyses provide complementary image-level and feature-level perspectives on model behavior while remaining subject to the limitations of post hoc explainability methods.

## 5. Discussion

This study investigated the effectiveness of transformer-based deep learning and hybrid feature fusion for the classification of paratuberculosis from histopathological tissue images. Across stratified five-fold cross-validation, all three Swin-based configurations achieved strong discriminatory performance, with mean ROC–AUC values ranging from 0.977 to 0.979. The standalone end-to-end Swin-Tiny model achieved the highest mean ROC–AUC of 0.979±0.015, whereas the Swin-embedding XGBoost and Hybrid Swin-Texture models achieved the highest mean accuracy of 0.928. These findings demonstrate that transformer-derived representations can effectively capture discriminative information from paratuberculosis histopathological images despite the relatively limited size of the available dataset. The results are consistent with recent studies demonstrating the effectiveness of vision transformers in medical image analysis, where hierarchical attention mechanisms have shown strong capability for representing complex tissue morphology and contextual information [13,14,22,25].

The strong performance of the Swin-Tiny architecture may be associated with its hierarchical shifted-window attention mechanism. Unlike conventional CNNs, which progressively aggregate information through local convolutional receptive fields, the Swin Transformer models local image relationships within attention windows while enabling information exchange across neighboring windows through shifted-window attention [22]. Histopathological classification may depend on visual information represented at multiple spatial scales, including cellular appearance, inflammatory patterns, tissue organization, and broader architectural changes. Similar observations have been reported in computational pathology, where transformer-based architectures have demonstrated strong performance by modeling both localized image characteristics and broader contextual relationships [14,20]. The hierarchical representation learned by Swin-Tiny may therefore have contributed to the consistently high classification performance observed in this study. However, because region-level pathological annotations were unavailable, the specific histological characteristics encoded by the learned representations could not be independently established.

The ablation analysis provided a more detailed assessment of the relative contributions of the learned and handcrafted feature representations. The GLCM/LBP-only XGBoost model achieved a mean ROC–AUC of 0.934±0.041, indicating that handcrafted texture descriptors contained independently discriminative information. Nevertheless, the texture-only model achieved lower overall performance than the configurations incorporating Swin-Tiny representations. Furthermore, adding the 38-dimensional GLCM/LBP representation to the 768-dimensional Swin embedding did not improve performance relative to the Swin-embedding XGBoost model. Both configurations achieved a mean accuracy of 0.928, recall of 0.915, specificity of 0.947, F1-score of 0.936, and ROC–AUC of 0.977. The two models also produced identical pooled confusion-matrix counts at the fixed classification threshold of 0.50.

The statistical analyses further indicated that the handcrafted texture descriptors did not provide a measurable incremental benefit after the Swin representations had been incorporated. The hybrid model produced a pooled ROC–AUC difference of −0.00084 relative to the Swin-embedding XGBoost model, with a paired bootstrap 95% confidence interval of [−0.00214,0.00020]. Because the confidence interval included zero and the magnitude of the difference was negligible, the results did not support improved discriminatory performance from the addition of the GLCM/LBP descriptors. McNemar’s exact test similarly showed no difference in paired classification correctness between the two configurations at the fixed threshold of 0.50 (p=1.000). These findings indicate that, although the handcrafted texture features were independently informative, much of their predictive information may already have been represented within the learned Swin-Tiny embedding space.

The feature-importance analysis was consistent with the ablation and statistical findings. Swin-derived embedding features accounted for 99.4% of the aggregate gain across the hybrid XGBoost models, whereas the combined GLCM/LBP descriptors accounted for 0.6%. In addition, no handcrafted GLCM or LBP descriptor appeared among the 20 highest-ranked individual predictors. These results suggest that the hybrid classifier relied predominantly on the learned deep representation. The difference in feature-family dimensionality should nevertheless be considered when interpreting aggregate importance because the Swin representation contained 768 dimensions, whereas the handcrafted representation contained 38 features. Moreover, gain-based importance reflects how frequently and effectively features contribute to decision-tree splits and should not be interpreted as a direct measure of biological relevance. Taken together with the ablation results, however, the findings do not support the original assumption that handcrafted texture descriptors provide substantial complementary predictive information when combined with Swin-Tiny embeddings in the present dataset.

The present findings are consistent with previous research on automated paratuberculosis classification while extending the literature through the evaluation of transformer-derived and handcrafted feature representations. Şengöz et al. [16] demonstrated that image preprocessing using contrast-limited adaptive histogram equalization improved VGG-16 classification performance, while Yiğit et al. [17] used Grad-CAM to improve the interpretability of CNN-based classification. In contrast to these convolutional approaches, the present study evaluated a hierarchical Swin Transformer and systematically compared end-to-end classification, handcrafted texture features, Swin embeddings with XGBoost, and a fused Swin-texture representation. The results indicate that the transformer-derived representations were the principal source of predictive performance and that the more complex hybrid representation did not provide a measurable advantage over the Swin-based alternatives. This finding is relevant because it demonstrates the importance of evaluating feature-fusion architectures through explicit ablation rather than assuming that the addition of handcrafted descriptors necessarily improves model performance.

Beyond the paratuberculosis literature, the results align with broader developments in computational pathology. Vision transformers have emerged as competitive approaches for histopathological image analysis because they can represent localized image characteristics while progressively integrating information across larger spatial regions [22,25]. Hybrid approaches combining deep representations with traditional machine-learning classifiers may also provide useful alternatives when annotated datasets are limited [20,27]. In the present study, the Swin-embedding XGBoost model achieved strong classification performance, but the addition of GLCM/LBP descriptors did not further improve discrimination. Therefore, the value of hybrid feature fusion may depend on the dataset, imaging characteristics, feature design, and extent to which handcrafted descriptors provide information not already encoded by the deep representation.

An additional contribution of this work is the use of complementary image-level and feature-level explainability analyses. Grad-CAM provided qualitative visualizations of the image regions associated with the predictions generated by the standalone Swin-Tiny model. The representative MAP-positive and MAP-negative examples exhibited spatially distributed activation patterns, indicating that the model responses were associated with multiple image regions rather than a single fixed location. However, the highlighted regions were not independently compared with pathologist-provided region-level annotations. The Grad-CAM visualizations, therefore, cannot establish that the activated regions correspond to specific MAP-associated lesions, granulomatous structures, or other biologically validated pathological characteristics. Similarly, the XGBoost gain analysis identified the relative contribution of the learned and handcrafted feature representations but did not provide direct biological interpretations of the individual embedding dimensions. These explainability analyses should consequently be interpreted as post hoc assessments of model behavior rather than evidence of pathological causality or clinical validity [17,31,32].

Overall, the revised findings demonstrate that Swin-Tiny representations provide strong discriminatory information for image-level paratuberculosis histopathology classification. Although the handcrafted GLCM/LBP descriptors exhibited independent predictive capability, their inclusion did not improve performance beyond the Swin embeddings. The principal contribution of the hybrid analysis is therefore not evidence of superior performance through feature fusion, but an empirical assessment of the relative and incremental value of learned and handcrafted representations within a controlled cross-validation framework. These findings support further investigation of transformer-based methods in veterinary computational pathology while emphasizing the need for independent external validation before considering diagnostic or decision-support applications.

## 6. Limitations

Several limitations should be acknowledged. The study used a relatively small publicly available dataset obtained from a single source. Following duplicate screening, the final analytical cohort contained 349 unique histopathological images, comprising 199 MAP-positive and 150 MAP-negative samples. Although stratified five-fold cross-validation enabled all eligible images to contribute to held-out evaluation, internal cross-validation does not establish generalizability to images obtained from other institutions, laboratories, animal populations, staining protocols, microscopes, or digital imaging systems. The reported findings should therefore be interpreted as internal image-level cross-validation performance rather than evidence of external diagnostic validity.

An additional limitation concerns the absence of unique animal, slide, and specimen identifiers in the available dataset documentation, image filenames, and directory structure. Consequently, biological grouping at the animal, slide, or specimen level could not be established, and it was not possible to independently determine whether multiple images originated from the same biological source. The cross-validation partitions were therefore constructed at the image level after exact- and near-duplicate screening. Although duplicate screening reduced the risk of identical or highly similar images appearing across partitions, it cannot exclude dependence arising from different fields of view obtained from the same animal, tissue specimen, or histological slide. Future datasets should provide animal-level, specimen-level, and slide-level identifiers to support group-aware partitioning and a more rigorous assessment of generalization.

The available dataset also lacked sufficiently detailed metadata regarding scanner hardware, magnification, staining batch, tissue-processing conditions, and image-acquisition protocols. Reinhard stain normalization was implemented using fold-specific target statistics derived exclusively from each outer training partition to reduce color variation without introducing information from held-out images. Nevertheless, stain normalization cannot account for all sources of inter-laboratory or acquisition-related variability. External evaluation using independently collected images from different laboratories and acquisition settings is required to determine whether the observed performance is maintained under domain shift.

The present study focused on binary image-level classification of MAP-positive and MAP-negative histopathological images because expert-annotated lesion types, disease stages, severity grades, and region-level pathological annotations were unavailable. Although binary classification may support the identification of image patterns associated with paratuberculosis, the disease can exhibit heterogeneous lesion characteristics and varying degrees of pathological severity. More clinically informative systems may require the ability to grade lesion severity, characterize disease progression, distinguish among histopathological lesion patterns, or differentiate paratuberculosis from other inflammatory and granulomatous intestinal diseases. Developing and evaluating such systems will require larger datasets with diagnostic labels and region-level annotations established or independently verified by experienced veterinary pathologists.

The explainability analyses should also be interpreted with caution because they were exploratory and post hoc. Grad-CAM identified spatial regions associated with model predictions but did not establish that the highlighted regions represented biologically meaningful or diagnostically relevant tissue structures. The activation maps were not quantitatively compared with expert-defined lesion annotations, and no formal localization analysis was possible. Similarly, XGBoost gain-based importance described feature use within the fitted classifier but did not establish causal or biological relevance. Future studies should incorporate pathologist-annotated regions of interest and quantitative localization measures to assess whether model attention corresponds to established histopathological characteristics.

The absence of a measurable performance improvement from adding GLCM/LBP descriptors should be interpreted within the context of the present dataset and feature design. The findings do not imply that handcrafted texture features are universally uninformative. Alternative texture representations, stain-specific features, multiscale descriptors, or feature-selection strategies may provide complementary information in larger or more heterogeneous datasets. Future research should evaluate these possibilities using group-aware development cohorts and independent external validation datasets.

## 7. Conclusions

This study evaluated an explainable framework for automated paratuberculosis classification from histopathological tissue images using a pretrained Swin-Tiny Transformer, handcrafted GLCM/LBP texture descriptors, and XGBoost classification. The results demonstrated strong performance across the Swin-based configurations, with the standalone Swin-Tiny model achieving the highest mean ROC–AUC.

The ablation and statistical analyses showed that the handcrafted texture descriptors contained independently discriminative information but did not provide a measurable improvement when combined with the Swin-Tiny embeddings. Feature-importance analysis further indicated that the hybrid classifier relied predominantly on the learned Swin representations. Grad-CAM provided qualitative insight into image regions associated with model predictions but should not be interpreted as confirmation of biological relevance.

Future research should validate these findings using larger, multi-institutional datasets with animal-, specimen-, and slide-level identifiers. External evaluation across different laboratories, staining protocols, imaging systems, and animal populations will be necessary to assess the generalizability and potential diagnostic utility of the approach.

## Figures and Tables

**Table 1:** Summary of related studies and comparison with the proposed framework.

Study	Domain	Method	Main Finding	Gap
Şengöz et al. [16]	MAP histopathology	CLAHE + VGG-16	F1-score improved from 93% to 98%.	No XAI or transformer models.
Yiğit et al. [17]	MAP histopathology	CNN + Grad-CAM	Accurate and explainable lesion detection.	CNN-only architecture.
Wang et al. [19]	Chest CT imaging	3D-ResNet + CAM	Differentiated nontuberculous mycobacterial with an AUC of 0.86.	Not MAP-specific and not histopathology.
Hananeh et al. [35]	MAP histopathology	Expert-annotated dataset	Provided benchmark image dataset.	No predictive model.
Lee et al. [36]	MAP microbiome	Machine learning	Identified microbial biomarkers of infection.	Not image-based.
Imada et al. [18]	Johne’s disease	Tree-based ML	Predicted future ELISA outcomes.	No histopathology analysis.
Yaman et al. [37]	MAP genomics	Machine learning	Identified genetic susceptibility markers.	No image data.
Komura and Ishikawa [38]	Histopathology	DL review	Established DL potential in pathology.	Not MAP-specific.
Proposed Study	MAP histopathology	Swin-Tiny + texture features + XGBoost + XAI	Hybrid transformer–texture framework with explainability.	Addresses the lack of interpretable transformer-based hybrid models for MAP diagnosis.

**Table 2:** Summary of the dataset-integrity screening process and final analytical cohort.

Dataset Stage	MAP-Positive	MAP-Negative	Total
Original public dataset	209	157	366
Exact duplicates removed	10	6	16
Verified near-duplicates removed	0	1	1
Final analytical cohort	199	150	349

**Table 3:** Summary of the image preprocessing and augmentation procedures used in the revised experiments.

Procedure	Application	Parameter or Implementation	Value
Reinhard normalization	All partitions	Color space	CIELAB
		Target statistics	Mean of per-image channel means and standard deviations from the outer training partition only
		Numerical stability constant	$\epsilon =10^{-6}$
Random resized crop	Swin fine-tuning: training only	Output size; scale; aspect ratio	224×224; 0.80–1.00; 3/4–4/3
Horizontal flip	Swin fine-tuning: training only	Application probability	0.50
Vertical flip	Swin fine-tuning: training only	Application probability	0.50
Rotation	Swin fine-tuning: training only	Angular range	$-20^{\circ }$ to $+20^{\circ }$
Color jitter	Swin fine-tuning: training only	Brightness; contrast; saturation; hue	0.15; 0.15; 0.10; 0.02
Deterministic resize	Inner validation, outer test, and feature extraction	Output size	224×224
ImageNet normalization	All Swin inputs	Channel means	(0.485,0.456,0.406)
		Channel standard deviations	(0.229,0.224,0.225)

**Table 4:** Model configurations evaluated in the ablation analysis.

Model Configuration	Input Representation	Classifier	Purpose
Standalone Swin-Tiny	Reinhard-normalized histopathological images	Swin-Tiny classification head	Evaluates end-to-end classification using the transformer architecture
GLCM/LBP + XGBoost	Handcrafted GLCM and LBP texture descriptors	XGBoost	Measures the independent predictive value of the handcrafted texture representation
Swin embeddings + XGBoost	768-dimensional Swin-Tiny embeddings	XGBoost	Evaluates the predictive value of the learned deep representation using a common tree-based classifier
Swin + GLCM/LBP + XGBoost	Concatenated Swin-Tiny embeddings and handcrafted texture descriptors	XGBoost	Measures the incremental contribution of handcrafted texture features beyond the Swin representation

**Table 5:** Ablation performance of the four model configurations evaluated using stratified image-level five-fold cross-validation. Values are reported as mean ± standard deviation across the five outer folds. The highest mean value for each metric is shown in bold.

**Model configuration**	**Accuracy**	**Precision**	**Recall**
GLCM/LBP + XGBoost	0.865±0.038	0.882±0.050	0.884±0.029
Swin-Tiny (end-to-end)	0.920±0.036	0.942±0.061	0.920±0.021
Swin embeddings + XGBoost	0.928±0.030	0.959±0.039	0.915±0.022
Swin + GLCM/LBP + XGBoost	0.928±0.027	0.958±0.030	0.915±0.022
**Model configuration**	**Specificity**	**F1-score**	**ROC–AUC**
GLCM/LBP + XGBoost	0.840±0.076	0.883±0.031	0.934±0.041
Swin-Tiny (end-to-end)	0.920±0.087	0.930±0.029	0.979±0.015
Swin embeddings + XGBoost	0.947±0.051	0.936±0.027	0.977±0.016
Swin + GLCM/LBP + XGBoost	0.947±0.038	0.936±0.024	0.977±0.017

**Table 6:** Paired statistical comparisons of model performance using pooled out-of-fold predictions. ROC–AUC differences are reported as the second model minus the first model. Confidence intervals were estimated using paired bootstrap resampling with 2000 iterations. McNemar’s exact test evaluated paired classification outcomes at a fixed probability threshold of 0.50.

Model Comparison	ROC–AUC Difference	95% CI	McNemar *p*-Value
Swin embeddings + XGBoost vs. Hybrid	−0.00084	[−0.00214,0.00020]	1.000
Standalone Swin-Tiny vs. Hybrid	−0.00050	[−0.0105,0.0091]	0.549
GLCM/LBP + XGBoost vs. Hybrid	+0.0460	[0.0248,0.0709]	<0.001

## Data Availability

The original histopathology dataset is publicly available from the Mendeley Data repository published by Hananeh et al. [35]. To respect the ownership and provenance of the original dataset, the processed images are not redistributed. The preprocessing scripts, duplicate-screening metadata, image manifests, cross-validation fold assignments, model training code, and other supporting materials used to generate the analytical cohort are available from the corresponding author upon reasonable request.

## Abbreviations

The following abbreviations are used in this manuscript:

AI	Artificial Intelligence
AUC	Area Under the Curve
CNN	Convolutional Neural Network
FN	False Negative
FP	False Positive
FPR	False Positive Rate
GLCM	Gray-Level Co-occurrence Matrix
Grad-CAM	Gradient-weighted Class Activation Mapping
H&E	Hematoxylin and Eosin
LBP	Local Binary Pattern
MAP	*Mycobacterium avium* subsp. *paratuberculosis*
ML	Machine Learning
MLP	Multi-Layer Perceptron
ROC	Receiver Operating Characteristic
ROC-AUC	Receiver Operating Characteristic Area Under the Curve
SW-MSA	Shifted Window Multi-Head Self-Attention
TN	True Negative
TP	True Positive
TPR	True Positive Rate
ViT	Vision Transformer
W-MSA	Window Multi-Head Self-Attention
XAI	Explainable Artificial Intelligence
XGBoost	Extreme Gradient Boosting
